# Droplet Velocity Measurement Based on Dielectric Layer Thickness Variation Using Digital Microfluidic Devices

**DOI:** 10.3390/bios8020045

**Published:** 2018-05-08

**Authors:** Siti Noor Idora Syafinaz Zulkepli, Nor Hisham Hamid, Vineeta Shukla

**Affiliations:** Department of Electrical and Electronic Engineering, Universiti Teknologi PETRONAS, Seri Iskander 32610, Malaysia; hishmid@utp.edu.my (N.H.H.); vineeta.banasthali@gmail.com (V.S.)

**Keywords:** digital microfluidic biochips, electrowetting-on-dielectric, printed circuit board, droplet actuation

## Abstract

In recent years, the number of interdisciplinary research works related to the development of miniaturized systems with integrated chemical and biological analyses is increasing. Digital microfluidic biochips (DMFBs) are one kind of miniaturized systems designed for conducting inexpensive, fast, convenient and reliable biochemical assay procedures focusing on basic scientific research and medical diagnostics. The role of a dielectric layer in the digital microfluidic biochips is prominent as it helps in actuating microliter droplets based on the electrowetting-on-dielectric (EWOD) technique. The advantages of using three different material layers of dielectric such as parafilm, polytetrafluoroethylene (PTFE) and ethylene tetrafluoroethylene (ETFE) were reported in the current work. A simple fabrication process of a digital microfluidic device was performed and good results were obtained. The threshold of the actuation voltage was determined for all dielectric materials of varying thicknesses. Additionally, the OpenDrop device was tested by utilizing a single-plate system to transport microliter droplets for a bioassay operation. With the newly proposed fabrication methods, these dielectric materials showed changes in contact angle and droplet velocity when the actuation voltage was applied. The threshold actuation voltage for the dielectric layers of 10–13 μm was 190 V for the open plate DMFBs.

## 1. Introduction

Microfluidic technology has been introduced as a part of lab-on-a-chip (LOCs) devices, in which several laboratory functions are integrated on a single chip [1,2]. Microfluidic technology has the ability to manipulate small volumes of samples or reagents from µL to pL range [3,4]. As microelectromechanical systems (MEMS) are scaled down, microfluidic technology becomes prominent in various medical diagnostics processes [3,5]. Microfluidic technology has the potential of replacing macroscale machines like biochemical analyzers in medical applications [6,7]. Microfluidic technology can be approached in two ways, i.e., continuous or digital. The physical structure of continuous flow-based microfluidic is less complex, as the liquid flow through channel and an additional hardware of micro valves, micro pumps are required to manipulate the liquid [6]. Unlike continuous flow microfluidics, the development of digital microfluidic biochips (DMFBs) is very advantageous in term of its feasibility, where only discrete droplets are manipulated independently to realize various liquid operations [8].

There are many applications of DMFBs in biochemistry, biotechnology and bioassays due to their small features. For example, in bioassays, clinical diagnostic analyses for glucose assays, blood sample preparation, and drug synthesis can be conducted [6,9], thanks to the flexibility, programmability, and reduced wastage of sample in digital microfluidic biochips [10,11]. Various liquid operations such as detection, actuation, merging, and separation of droplets can be performed on a DMFB [2,12]. A digital microfluidic biochip consists of a movable droplet on top of the electrode surface which is known as electrowetting [13]. The droplet behavior is determined by interfacial tensions as three-phase forces of liquid, solid and gas is pulled towards each contact line as denoted by Young’s equation:

(1) $$cos_{\theta_{Y}}=\frac{\sigma_{sg}-\sigma_{sl}}{\sigma_{lg}},$$

where $\theta_{Y}$ is the Young’s contact angle, $\sigma_{sg}$ is interfacial tension between solid–gas, $\sigma_{sl}$ is interfacial tension between solid–liquid, and $\sigma_{lg}$ is interfacial tension between liquid–gas [14,15]. The existence of an applied voltage to the electrode surface builds up an electric double layer between the dielectric and droplet interface, which will decrease the droplet contact angle [16]. However, the thickness of the dielectric layer, the dielectric material and the limit of applied voltage on the electrodes were major concerns for researchers working in the fabrication of DMFB devices over the past few years [17].

For EWOD, dielectric polarization takes place when voltage is applied at the bottom of the electrode. The effect of dielectric polarization creates an internal electric field which induces a charge accumulation on the droplet surface. Hence, the distribution of accumulating charges will create an electrostatic force acting on the droplet, which leading the droplet toward the active electrode [16]. The electrostatic force acting on the droplet is obtained by integrating the variables as follows:

(2) $$F_{d}=\int S_{12}+S_{13}T\cdot n\;dS,$$

where $F_{d}$ is the electrostatic force, $S_{12}$ and $S_{13}$ are the subscripts of the droplet, surrounding fluid and dielectric layer, respectively, whereas, T is the Maxwell stress tensor. The forces due to the free electric charges and the forces due to the polarization of the material is known as Maxwell stress tensor [16]. An electrostatic force distribution increases due to the charge distribution near the contact line of three-phase forces.

However, to facilitate the movement of the droplet, a suitable thickness of the dielectric layer is necessary [18,19]. The electrode size also plays an important role in actuating the droplet because the top plate is absent in an open system. This research focused on determining the required applied voltage for the actuation of droplet with different dielectric materials. The dielectric thickness is the main key for droplet actuation because thinner layer of dielectric provides faster droplet actuation [8,20]. Nevertheless, a thin dielectric layer is susceptible to dielectric breakdown due to high voltage [2,18]. Thus, the required applied voltage for droplet actuation is dependent on the thickness of dielectric layer.

This paper presented the fundamental and experimental results of minimum applied voltage required to actuate the droplet using three dielectric materials such as parafilm, PTFE and ETFE. The material thickness would affect the droplet actuation. The velocity of the actuated droplet was measured experimentally. The paper was organized as follows. The related works detailing the dielectric materials used for the fabrication of digital microfluidic biochips (DMFBs) were reviewed in Section 2. The new methodology for fabricating the DMFB and the required applied voltage as a function of dielectric layer thickness were highlighted in Section 3. Then, the experimental results of contact angle and droplet velocity for all types of dielectric layers were reported in Section 4. Finally, the work was concluded and some future works were recommended in Section 5.

## 2. Related Work

**Figure 1:**
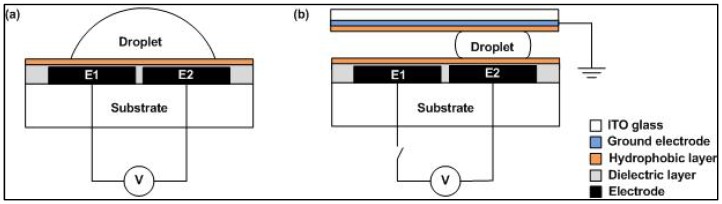
Types of digital microfluidic biochip (DMFB) devices: (**a**) open system; (**b**) closed system.

Electrowetting-on-dielectric (EWOD) works based on a droplet actuation mechanism that involves surface tension force between liquid and solid [21]. There are two types of EWOD digital microfluidic devices, i.e., open and closed systems [6]. Figure 1 depicts the difference between these two systems, where the top plate is found in closed DMFB systems only [22].

In the closed system, the droplet is confined between two parallel plates with indium tin oxide (ITO) glass as the top plate. The droplet evaporation rate can be reduced by developing this system. Here, the top plate acts as a ground electrode. The bottom layers of open and closed systems consist of four layers: a substrate, an electrode array, a dielectric layer and a hydrophobic layer [23]. The electrode layer is an array of cells that forms a pathway for the droplet movement [23]. The hydrophobic layer is coated on the top and bottom plates in order to decrease the surface wettability [24]. Meanwhile, the dielectric layer is used to maximize the electrostatic force acting on the droplet is based on Equation (2) [13]. As shown in Figure 1a, the droplet moves to electrode E2 by grounding the electrode E1 and activating the electrode E2. The actuation mechanisms of open and closed DMFB systems are similar.

EWOD is believed to be an efficient technique for droplet actuation [17,25]. High droplet speed can be attained in geometrically-simple electrode arrays [10,21]. A charge is built up at the surface between the solid dielectric layer and the liquid interface when the voltage is applied at the bottom of E2. Electric field is created which changes the interfacial surface energy and enables the movement of droplet from E2 to E1 as shown in Figure 1. The thickness of dielectric layer is the main key in droplet actuation as the change in contact angle depends on the thickness of dielectric layer and the type of hydrophobic material used [25]. The dielectric layer affects the total capacitance based on Equation (3):

(3) $$C=\frac{k\epsilon_{0}A}{t_{d}}$$

where *C* is the total capacitance, *k* is the dielectric constant, $\epsilon_{0}$ is the permittivity of free space, *A* denotes the area, and $t_{d}$ is the dielectric layer thickness. By adding the dielectric layer, the capacitance values of the DMFB cell is decreased.

In literature, various substrates have been used in developing the DMFB devices [11,20,23,26]. Commonly, glass and silicon have been used as the substrate material [23,27]. There is a rising demand for having a substrate material that is low cost and easy to fabricate (without accessing the clean room). For example, in paper-based microfluidics, paper is the most common substrate used for the screen printing of electrodes on top of paper [28]. Besides paper-based substrates, polyimide foil has also been used as a substrate, replacing the expensive glass and silicon. However, for inkjet printing technique, the chance of stain spreading on the printed device from the chemical reagent is higher [29]. Therefore, this technique is only limited to certain types of chemical reagent. For laser-printed polyester film, the microfluidic channel is formed by printing the device on a transparency film which is limited for continuous flow microfluidics [30].

Recently, printed circuit boards (PCB)-based DMFB fabrication technique has been studied [11,20,23,27]. The low cost PCB substrate is more durable and flexible than glass and silicon, which involves costly photolithography process [19]. Additionally, the cost of fabrication is also reduced by replacing the glass substrate with the PCB substrate [23]. The first step in the fabrication of PCB-based DMFB includes designing and editing of the intended circuit based on the required application [31]. In the editing step, design rules check (DRC) is necessary before generating the Gerber files and plotting the film. Next, brushing and cleaning operations can be performed after running the CNC drilling. The plotted film is exposed to the UV light after laminating the dry film. Thereafter, the film is developed by chemical wet process after exposing it for some time. Then, the film is etched in order to remove any unnecessary deposited chemical. Finally, electrical testing is conducted before the surface treatment process [27]. Therefore, the simplest fabrication procedure of DMFB is beneficial in realizing the bioassay operation. For patterning the electrodes in DMFBs, various materials such as gold, ITO, chromium, carbon and copper have been used [19,23]. The performance is not dependent on the electrode material. Nevertheless, the actuation of the droplet is dependent on the electrode pattern [14]. Unlike electrodes, the choice of dielectric material is significantly important when designing a DMFB device [19] as it would affect the characteristics of a DMFB device via the dielectric constant.

**Figure 2:**
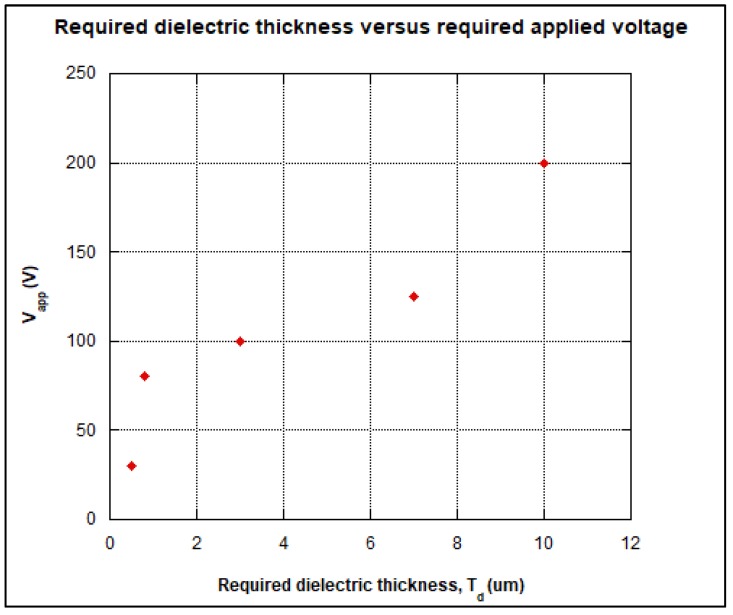
The required applied voltage corresponding to the dielectric thickness [3].

Basically, a high dielectric constant materials is preferred in actuating the droplet for bioassay operation as the required applied voltage can be reduced [32]. However, a high voltage is required for droplet transportation, mixing and separation in DMFB [25]. Thus, a high dielectric constant is not recommended for this work because when high voltage is applied the dielectric layer would undergo breakdown according to Equation (4):

(4) $$V_{app}=\frac{Qt_{d}}{k\epsilon_{0}A}$$

where $V_{app}$ denotes the applied voltage, *k* is the dielectric constant, $\epsilon_{0}$ is the permittivity of free space, *A* is the area of electrodes, and $t_{d}$ is the dielectric thickness. The required applied voltage $V_{app}$ can also be interpreted as an actuation threshold voltage which is determined by the dielectric thickness to dielectric constant ratio [13]. Figure 2 shows the actuation threshold voltage increases with respect to the ratio of dielectric thickness in order to actuate the droplet for EWOD mechanism as reported in [3]. However, the most available dielectric thickness to be found is more than 12 μm. As the thickness of dielectric layer is increased, the required applied voltage is increased as well.

These factors are normally taken into account while designing a DMFB device [13,24]. Previous studies have considered SiO_2_ as the dielectric material, due to its low dielectric constant. A minimum of 80 V of threshold voltage is required for droplet transportation in DMFB. In order to overcome the surface tension between the droplet and the dielectric layer, the required voltage should be increased during the mixing and splitting operations [17]. Typically, the dielectric layer can be coated via various techniques such as vapor deposition, sputtering, vacuum evaporation and spin coating. Again, the device behavior is heavily dependent on the choice of the coating technique. One of the prominent problems is called the stoichiometry control problem, which occurs when an incorrect ratio of silicon and oxygen is used for silicon oxide reaction. Therefore, the composition of dielectric material can significantly affect the electrical characteristics of DMFB device [33]. The easiest deposition technique is spin coating, in which an accurate amount of coating liquid is placed as an insulation layer. Low cost dielectric materials such as parafilm, PTFE and ETFE were used in the current work. The dielectric constants of several common materials which can be employed in EWOD device are summarized in Table 1.

The final layer of DMFB device is the hydrophobic layer, which is designed to reduce the surface energy by facilitating droplet actuation [24]. Fluoropolymer-based materials such as Teflon and Cytop were used for fabricating the hydrophobic layer [13,18,23]. The reported contact angle ranged from 105° to 117°.

However, when cooking oil was used to replace the expensive fluoropolymer-based materials, the contact angle was reduced to 74° [14]. The promising stabilization of fluoropolymer-based materials is more prominent than that of the cooking oil [14]. The promising stabilization of fluoropolymer-based materials is more prominent than that of the cooking oil [3,23]. However, fluoropolymer-based materials are typically not vastly available and expensive, making cooking oil or silicon oil the best replacement for hydrophobic material. In this paper, the droplet velocities for cases employing different dielectric materials were measured and compared in order to obtain the required actuation voltage. The droplet wettability was observed for all dielectric materials. The interaction between dielectric surface and liquid would change the surface energy, leading to droplet actuation. A simple method for calculating droplet velocity was proposed as well.

## 3. Our Approach

**Figure 3:**
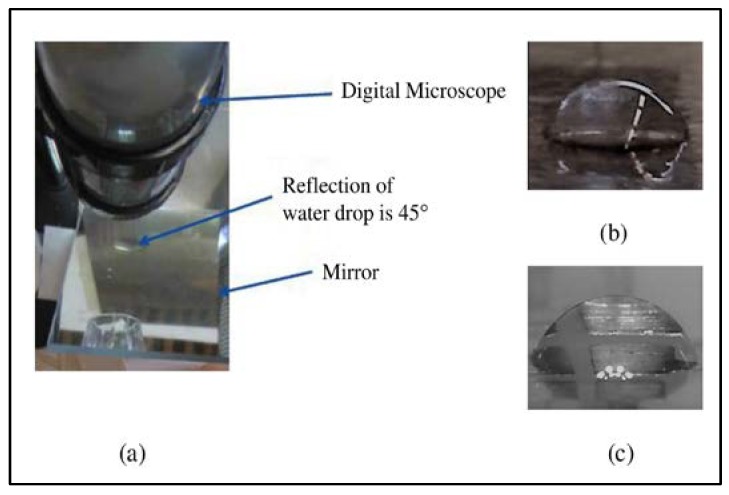
(**a**) Side view contact angle measurement. Change in contact angle: (**b**) first prototype and (**c**) second prototype [14]. Reproduced with permission from Siti Noor Idora Syafinaz Zulkepli, Nor Hisham Hamid and Vineeta Shukla, A low cost open droplet-based microfluidic devices on printed circuit board; published by IEEE, 2017.

In this section, a simple method used to measure the contact angle of tested droplet is given. There are several methods available in coating the dielectric and hydrophobic layers, as explained in the upcoming section. The wetting phenomenon of tested droplet can be observed by measuring the contact angle of stationary droplet for the EWOD technique given by [35]:

(5) $$\frac{\theta }{2}=tan^{-1}(h/r)$$

where θ represents the contact angle, *h* is the height of apex, and *r* represents the droplet radius [36]. Generally, the contact angle can be measured in either side view or top view. Based on this equation, the side view method is more approachable as shown in Figure 3 as the top view method does not work on hydrophobic surfaces [35].

A 2.5 μL of dionized (DI) water was placed on the electrode. PTFE and cooking oil layers were used as dielectric and hydrophobic layers as reported in [14]. A change in contact angle from 80.59° to 69.36° is observed from the side view of digital microscope as shown in Figure 3b for first prototype of DMFB as reported in [14], while Figure 3c shows a change in contact angle from 75.42° to 73° for second prototype of DMFB as reported in [14]. Therefore, the wetting behavior can be characterized via contact angle calculation.

Various techniques have been applied to develop DMFB on different platforms such as printed circuit board, inkjet printing, laser printed polyester film and photosensitized PCB [36]. For the inkjet printing technique, the device is printed onto paper, which leads to chemical reagent smearing. Therefore, this technique is limited only to certain types of chemical reagent. For laser printed polyester film, the microfluidic channel is formed by printing the device on a transparent film which is limited to continuous flow microfluidics. Meanwhile, plastic-based substrate is used in photosensitized PCB technique; unfortunately, the plastic-based substrate is not easily available. In this work, we have used printed circuit board (PCB) to develop our DMFB device as PCB substrate is easily available and inexpensive [37].

**Figure 4:**
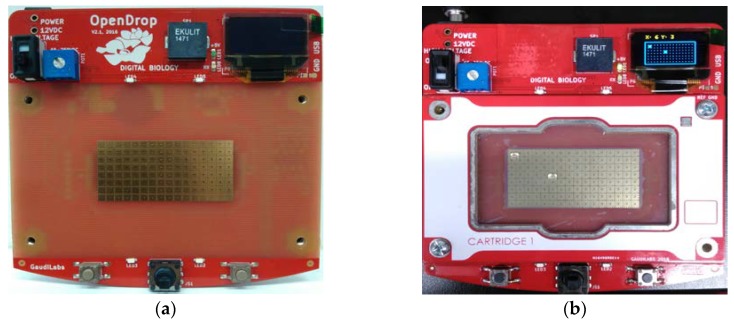
(**a**) OpenDrop device [3]; (**b**) 2 droplets of 2.5 μL size has been tested to observe the droplet movement.

In our previous work, we have fabricated a low cost open EWOD DMFB device on a standard FR4 printed circuit board (PCB). An array of 2 × 4 copper-based electrodes (3 × 3 mm^2^) has been fabricated with a separation distance (between electrodes) of 203.2 µm. Polyethylene film and cooking oil have been used as the materials for dielectric and hydrophobic layers, respectively. A 2.5 µL of DI water was tested in air by using the EWOD technique. Then, the contact angle was measured to observe the phenomenon of EWOD. In this work, we have used the OpenDrop device provided by Gaudi Labs [3]. This DMFB device is shown in Figure 4a.

In the current work, the EWOD DMFB was operated in air by using the OpenDrop device [3] to observe the threshold actuation voltage that can be applied on various dielectric materials. The electrodes were gold-coated and the DC to DC voltage regulator was integrated to actuate the droplet from 190 V to 330 V. As mentioned above, various methods of dielectric layer coatings are available in literature. However, the access to a clean room is quite challenging for certain researchers due to the lack of equipment and facilities [3,23]. Therefore, in order to overcome this problem, a simple and straightforward approach of dielectric layer coating is opted for based on the availability of the dielectric material. In this work, we have used three dielectric materials. The values of dielectric thickness (measured using digital vernier caliper) after performing hydrophobic coating are shown in Table 2. Additionally, the applied voltage required to actuate the droplet have been determined.

**Figure 5:**
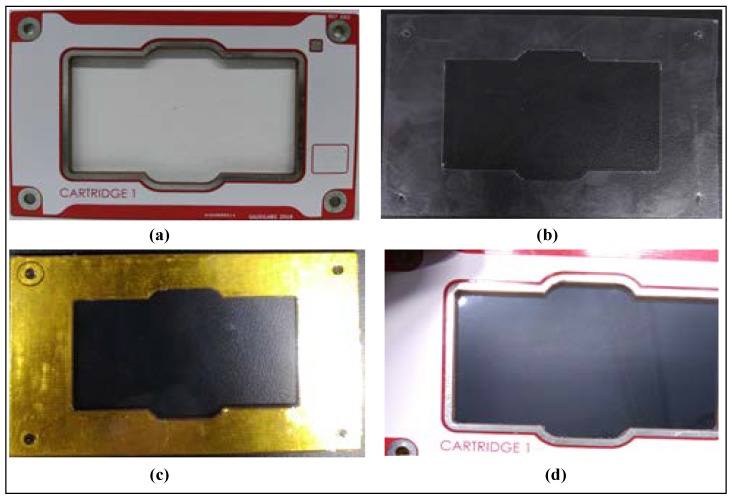
Preparing the dielectric layer coating: (**a**) frame without the dielectric coating; (**b**,**c**) kapton tape is used to stick the dielectric material; (**d**) frame with the dielectric layer.

Regarding the coating of materials such as parafilm, PTFE film and ETFE film considered in the current work, these materials must be properly stretched so that the film is tensed evenly. These films were stretched on a clean glass, and then cut so that they can fit nicely on the top of electrodes. In order to prevent film misplacement, a frame was developed for our coating process. A kapton tape was used to hold the film firmly at the back of the frame as shown in Figure 5.

The frame was then cleaned in the mixture of distilled water and isopropanol liquid using ultrasonic bath. After 15 min, the frame was rinsed with pure water and dried on the hot plate heated at around 80 °C.

In the dielectric film-coated frame, about 150 μL to 200 μL of fluoropel liquid was spun for 30 s at 3000 rpm. Then, the frame was dried on the hot plate heated from 80 °C to 150 °C for 20 min. This spin coating process is only necessary for fluoropel liquid. In the next phase, the hydrophobic layer was coated on top of the dielectric layer where silicon oil and cooking oil were used to prevent trapping of air bubbles inside the gap. Fluoropel liquid was used to reduce the surface energy between the droplet and the dielectric layer. These low-cost materials were attractive as they can be coated without accessing the clean room facilities. Unlike silicon oil and cooking oil, the spin coating process is necessary for coating the fluoropel liquid, as proper coating is needed.

The dielectric and hydrophobic layers were coated in order to actuate the stationary droplet for bioassay operation. A droplet can move easily to the adjacent electrodes when fluoropel is used as hydrophobic layer (instead of using silicon oil and cooking oil). The OpenDrop device was tested to observe the required applied voltage for the dielectric materials used such as parafilm, PTFE film and ETFE film as shown in Figure 4b.

## 4. Results and Discussions

### Measurement of Droplet Velocity

**Figure 6:**
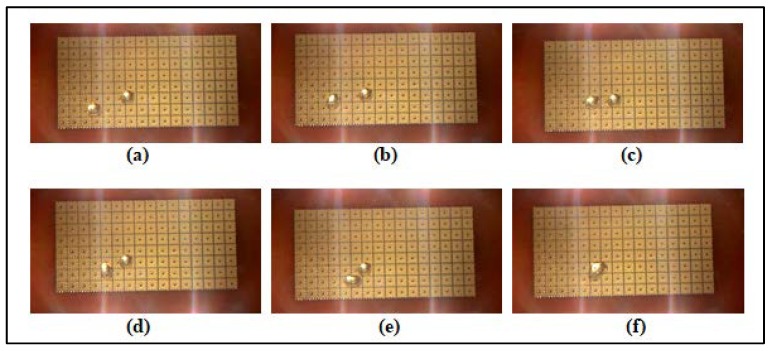
(**a**–**e**) The droplet is moving from one electrode array to the adjacent electrode array; (**f**) the droplet is merged together based on the captured video.

The droplet velocity was calculated via

(6) $$V=\frac{d_{AE}}{t_{AE}}$$

where $d_{AE}$ is the distance to reach the adjacent electrode, and $t_{AE}$ is the time taken to reach the adjacent electrode. For the applied voltage range of 190 V to 330 V, the velocity of droplet travelling across the 100 µm gap (between the electrodes) was calculated based on the captured video, as displayed graphically in Figure 6.

**Figure 7:**
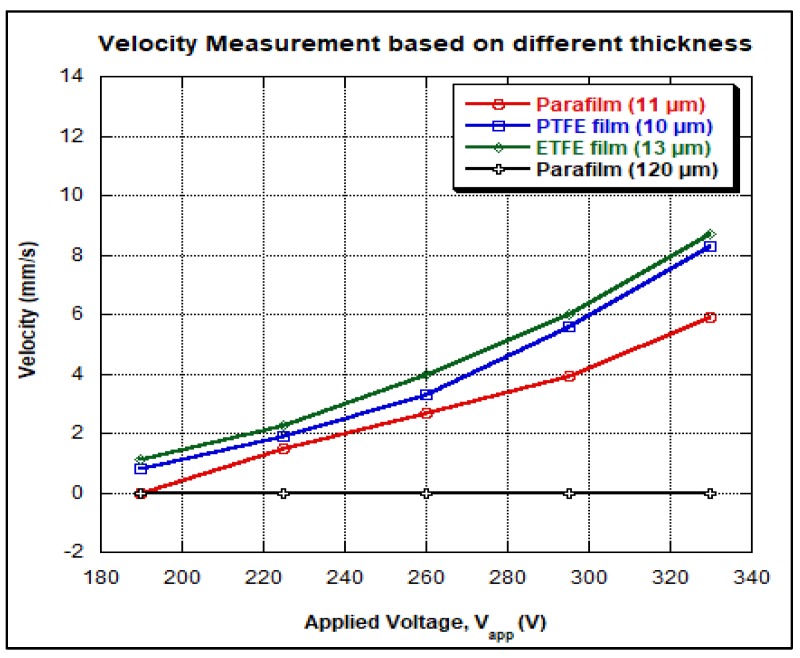
Experimental result for the droplet velocity measurement based on different dielectric materials.

In the experiments where parafilm was used as the dielectric layer material, the droplet was stagnant (dielectric layer thickness of 120 µm). However, when the applied voltage was increased from 190 V to 330 V, the droplet was able to move in the cases of PTFE film and ETFE film as shown in Table 2. As previously discussed, droplet motion could be started only when a suitable dielectric layer thickness is chosen and a proper minimum voltage is applied. By varying the thickness values of parafilm, PTFE film and ETFE film, both droplet velocity and applied voltage increase as shown in Table 3 and Figure 7.

For PTFE film, an average droplet velocity of 0.8 mm/s was observed when the voltage of 190 V was applied. Meanwhile, the droplet velocity of 1.23 mm/s was observed when the ETFE film was used. The droplet velocity using ETFE film was higher than that using PTFE film even though the thickness of PTFE film was less than 13 μm. This could be due to the fact that the fluoropel liquid used for hydrophobic coating of ETFE film was more durable as the frame was baked after spin coating. Besides the dielectric layer thickness, the coatings of dielectric and hydrophobic layers also affect the droplet velocity. While operating the OpenDrop device in air, the droplet speed was limited to 8.7 mm/s. On the other hand, the reported velocity of 80 mm/s was reported by the GaudiLabs team when the OpenDrop device was operated in the oil medium [3].

For this work, the unstretched parafilm was used because a thick parafilm (e.g., 120 μm) is unable to build a sufficiently strong electric field for moving the droplet. However, when the parafilm was stretched to 11 μm as shown in Figure 7, a significant droplet movement was observed. Therefore, these three different dielectric materials can be used to observe the velocity of the droplet. Based on the experimental results of OpenDrop device, the minimum DC voltage that can be applied is 190 V when the device is operated in air. The reliability of DMFB depends on the electrodes and the dielectric material used. Several electrodes became faulty after the DMFB device was operated repeatedly (due to breakdown of dielectric layer). Thus, for reliability purpose, the dielectric layer thickness and the dielectric material type should be chosen carefully. Also, droplet actuation is dependent on electrode size, electrode edges, and gap width between the inter electrode as reported in [14]. As shown in Figure 2, the threshold actuation voltage that can be applied is heavily dependent on the thickness of the dielectric material used.

## 5. Conclusions

The electrowetting-on-dielectric (EWOD) technique has been widely employed in droplet actuation for microfluidic applications. However, it requires a high operating voltage to realize droplet actuation. In order to alleviate this issue, the optimum thickness values of several types of dielectric layers have been identified. First, the surface wettabilities of cooking oil and PTFE have been observed by measuring the contact angles of DI water droplets on hydrophobic and dielectric layers, respectively. The results have indicated that for both cooking oil and PTFE layers, the contact angle decreased from 80.59 to 69.36° (measured from the side view method of contact angle measurement) as reported in [14]. The average droplet velocities in cases using parafilm, PTFE and ETFE dielectric layers of different thickness values have been investigated by varying the operating voltage. The current experiment has shown that the average droplet velocities in cases using parafilm (11 μm), PTFE (10 μm), and ETFE (13 μm) increased rapidly as the applied voltage increased from 190 V to 330 V. However, for unstretched parafilm of thickness 120 μm, no droplet movement has been observed, as the critical thickness of dielectric layer that causes droplet actuation should be less than 14 μm. Even though the thicknesses of both parafilm and PTFE were less than that of ETFE, the droplet velocity in ETFE was the highest (i.e., 8.7 mm/s) at 330 V operating voltage. This is because both dielectric and hydrophobic coatings play their roles in droplet actuation as well. Based on the experimental results, the dielectric layer of optimum thickness 11 μm with tolerable coating method has been recommended in order to reduce the operating voltage. Finally, the effect of the optimum thickness of the dielectric layer on the operating voltage in EWOD actuation has been tested and confirmed. This study contributes to the understanding of the effect of dielectric layer thickness on the droplet actuation. Also, a simple coating method has been proposed for future EWOD applications.

## Figures and Tables

**Table 1:** Various dielectric materials with dielectric constant [34]. PTFE: polytetrafluoroethylene.

Material	Dielectric Constant, *k*
FR4 PCB	4.8
Glass	5–10
Paper	3.85
Paraffin wax	2–2.4
Polyimide	2.25
PTFE (Teflon)	2.1
Silicon dioxide	3.9
Silicon	11.68
Vacuum	1

**Table 2:** Type of dielectric material used. ETFE: ethylene tetrafluoroethylene.

Type of Dielectric Materials	Thickness (µm)	Dielectric Constant, *k*
Parafilm	120	2.2
PTFE film	10	2.25
ETFE film	13	2.6

**Table 3:** Velocity measurement for various voltage values.

Applied Voltage, $V_{app}$ (V)	Type of Dielectric Materials			
	Parafilm (120 μm)	PTFE (10 μm)	ETFE (13 μm)	Parafilm (11 μm)
	Velocity (mm/s)			
190	0	0.8	1.23	0
225	0	1.9	2.25	1.5
260	0	3.3	4.0	2.7
295	0	5.6	6.0	3.9
330	0	8.3	8.7	5.9
